# The Impact of Persistent Noise Exposure under Inflammatory Conditions

**DOI:** 10.3390/healthcare11142067

**Published:** 2023-07-19

**Authors:** Inja Cho, Jeongmin Kim, Seungho Jung, So Yeon Kim, Eun Jung Kim, Sungji Choo, Eun Hee Kam, Bon-Nyeo Koo

**Affiliations:** 1 Department of Anesthesiology and Pain Medicine, Yonsei University College of Medicine, Seoul 03722, Republic of Korea; kdindin@yuhs.ac (I.C.); anesjeongmin@yuhs.ac (J.K.); jungshme@yuhs.ac (S.J.); kimsy326@yuhs.ac (S.Y.K.); natlis@yuhs.ac (E.J.K.); sjchu@yuhs.ac (S.C.); mirine1105@yuhs.ac (E.H.K.); 2 Anesthesia and Pain Research Institute, Yonsei University College of Medicine, Seoul 03722, Republic of Korea

**Keywords:** noise exposure, inflammation, neuroinflammation, cognitive impairment, anxiety-like behavior, blood–brain barrier

## Abstract

The aim of this study was to investigate the impact of noise exposure in an intensive care unit (ICU) environment on the development of postoperative delirium in a mouse model that mimics the ICU environment. Additionally, we aimed to identify the underlying mechanisms contributing to delirium and provide evidence for reducing the risk of delirium. In this study, to mimic an ICU environment, lipopolysaccharide (LPS)-injected sepsis mouse models were exposed to a 75 dB noise condition. Furthermore, we assessed neurobehavioral function and observed the level of neuroinflammatory response and blood–brain barrier (BBB) integrity in the hippocampal region. The LPS-injected sepsis mouse model exposed to noise exhibited increased anxiety-like behavior and cognitive impairment. Moreover, severe neuroinflammation and BBB disruption were detected in the hippocampal region. This study provides insights suggesting that persistent noise exposure under systemic inflammatory conditions may cause cognitive dysfunction and anxiety- like behavior via the mediation of BBB disruption and neuroinflammation. As a result, we suggest that the detailed regulation of noise exposure may be required to prevent the development of postoperative delirium.

## 1. Introduction

Delirium is a form of cognitive impairment that results in reduced attention span, agitation, and anxiety. Delirium rapidly manifests within a few days after surgery, and up to 80% of cases occur in the intensive care unit (ICU) [1,2]. Although recent advancements in critical care medicine have improved survival rates in the ICU, the impact of brain dysfunction associated with ICU stays is not completely understood [3,4]. The ICU is a challenging environment, as critically ill patients undergo intensive monitoring and invasive procedures, with inevitable yet considerable noise levels generated at all hours. We recently reported that (1) there are unavoidable severe noise levels in the ICU 24 h a day, (2) the noise levels are considerably higher than recommended levels, and (3) the noise occurs consistently throughout the day, with no differences between night and day [5]. The World Health Organization (WHO) guidelines recommend that the noise of hospital ward rooms should be restricted to A-weighted energy-equivalent sound pressure levels (SPLs) (in LAeq) and A-weighted maximum SPLs with fast time constants (in LAmax) of 30 and 40 dB, respectively [6]; however, the red alarm in the ICU, which signals fatal adverse events such as asystole and ventricular fibrillation, has a sound volume of 85 dB to alert medical staff. In addition, the WHO estimates that over 1 million healthy lives are lost every year in European countries alone due to environmental noise [7]. It has been determined that noise could cause negative health issues, such as annoyance [8], sleep disturbance [9], and cardiac vascular disease [10], and it is also suggested that annoyance and sleep disturbance have a profound effect on mental dysfunction [11,12,13]. Many studies have also emphasized the effects of noise on the impairment of the central nervous system (CNS) via increased oxidative stress, imbalance in neurotransmitter levels, inflammation, cognitive impairment, and delirium [4,14,15,16,17]. Furthermore, noise exposure is associated with alterations in DNA methylation [18] and telomere length [19]. These noise-induced changes are considered as biomarkers that can influence inflammatory and oxidative stress and can predict future diverse diseases, including cardiovascular diseases [20]. Recently, an animal study reported increased oxidative stress in rats that had been exposed to noise mimicking that in the ICU environment [21]. However, the pathogenetic mechanism underlying the way in which noise exposure is related to cognitive impairment has not been investigated in detail.

Inflammation is a complex biological response to harmful stimuli, including infection, tissue damage, and surgery [22]. In normal conditions, the blood–brain barrier (BBB) serves to protect neuronal cells while signaling the presence of systemic inflammation and infection to the brain to enable a protective sickness behavior response [23]. With increasing degrees of systemic inflammation or additional negative stimuli, the BBB becomes more permeable, and endothelial cell damage is induced [24]. As a result, these alterations result in brain dysfunction and disorder. 

Here, we investigated the impact of chronic noise exposure on the development of postoperative delirium in a lipopolysaccharide (LPS)-induced sepsis mouse model mimicking ICU environment, as well as the mechanism by which it occurs.

## 2. Materials and Methods

### 2.1. Animals

All in vivo experimental procedures were approved by the Institutional Animal Care and Use Committee of Yonsei University Health System (IACUC approval number, 2021-0097) certified by the Association for Assessment and Accreditation of Laboratory Animal Care International (AAALAC). Experimental mice were housed with free access to chow and water with a 12 h light/dark cycle in a pathogen-free facility at the Yonsei Biomedical Research Institute. We used male C57BL/6 mice aged 8–10 weeks (28–30 g) from Orient Bio (Seongnam, Gyeonggi-Do, Korea).

### 2.2. Experimental Design and Animal Modeling

**Figure 1:**
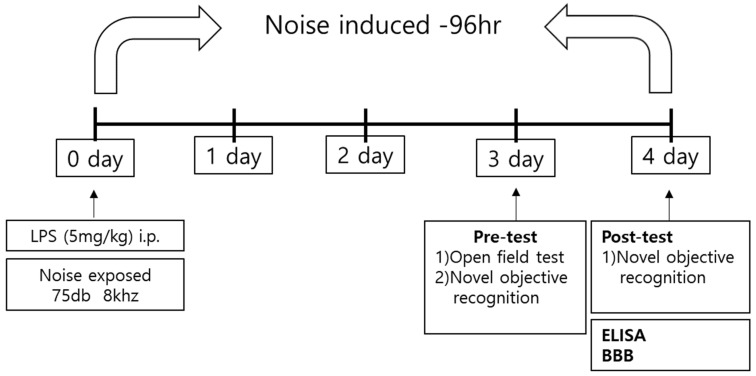
Schematic of the experimental procedure. Mice underwent behavioral tests 3 and 4 days after establishment of the LPS-induced sepsis model. After the behavioral test, mice were sacrificed, and the brains were harvested for experiments on inflammatory factors and BBB permeability. BBB, blood–brain barrier; LPS, lipopolysaccharide; ELISA, enzyme-linked immunosorbent assay.

We used an animal model of septic encephalopathy, our LPS-treated mouse model with induced systemic inflammation, to model patient conditions in the ICU (Figure 1). Many previous studies have modeled septic encephalopathy by administrating LPS intraperitoneally to mice at dosages ranging from 3 to 70 mg/kg [25,26,27,28,29,30]. In the present study, LPS (5 mg/kg) was administered to mice using intraperitoneal injection. The mice were randomly divided into the vehicle-treated group (sham control), LPS-treated group (LPS: 5 mg/kg), noise exposure group (75 dB noise), and LPS-treated + noise exposure group (LPS: 5 mg/kg + 75 dB noise).

Alarms on ICU monitors that alert medical staff of fatal adverse events, such as asystole and ventricular fibrillation, have a volume of 85 dB [2]. In addition, our previous clinical study confirmed that mechanical noise levels from a minimum of 41 dB to a maximum of 91 dB were measured in all ICU units [5]. Based on these results, we exposed mice to a moderate (stressful) noise level of 75 dB to mimic an ICU environment. A white noise generator (JEONGDO B&P) was used to generate 75 dB of mechanical noise. An amplifier connected to a loudspeaker was fixed at 20 cm above the mouse cage. This noise exposure mouse model was exposed to this noise for 4 days, because the average ICU stay is 2.8 days. Open-field tests (OFTs) were conducted on the 3rd day after noise stress exposure, whereas novel objective recognition tests (NORTs) were conducted on the 3rd and 4th days after noise exposure. During the neurobehavioral test, noise exposure was stopped for the duration. Upon completion of behavioral tests, the mice were sacrificed, and brain tissue was harvested.

### 2.3. Neurobehavioral Assessment

OFTs and NORTs were used for neurobehavioral assessment of the mice. The OFT was performed 3 days after noise exposure, whereas the NORT for cognition testing was performed 3 and 4 days after exposure.

The OFT has been frequently used to observe anxiety-like behavior in mice [31]. To perform the OFT, mice were placed in a square open-field arena (40 × 40 × 40 cm), and their behavior was recorded for 10 min. We assessed the total distance moved (to measure general activity and locomotor function) and the animal’s tendency to avoid the arena center (to measure anxiety-like behavior) 3 days after noise exposure.

The NORT was performed to evaluate cognition, specifically recognition memory [32]. During the familiarization phase (3 days after noise exposure), the mice were placed into a white box containing two identical objects (A + A), and they were allowed to explore for 10 min. During the test phase (4 days after exposure), each mouse was returned to the box with two objects, except one object was exchanged for a novel object (A + B), and the mouse was allowed to explore for 10 min. Time spent exploring each object was recorded during both phases. The discrimination index, which reflects cognitive ability, was evaluated as follows: (time spent exploring novel object B)/(time spent exploring novel B + familiar object A) × 100.

All neurobehavioral assessments were video-recorded and analyzed using an image analyzing system (SMART v2.5.21 software and SMART video Tracking system, Panlab Harvard Apparatus, Barcelona, Spain) by an assessor blinded to the treatment groups. After each test, the apparatus and objects were cleaned using 70% ethanol.

### 2.4. Enzyme-Linked Immunosorbent Assay (ELISA)

To obtain hippocampal brain tissue, mice were sacrificed 4 days after noise exposure, and the harvested hippocampal brain tissue was stored at −80 °C until use. To measure hippocampal levels of pro-inflammatory cytokines, such as interukine-1-beta (IL-1ß), IL-6, and tumor necrosis factor-alpha (TNF-α), and levels of BBB-integrity-related proteins, such as monocyte chemoattractant protein-1 (MCP-1), E-selectin, and P-selectin, brain tissues were lysed using a tissue protein extraction reagent (T-PER^®^ Tissue Protein Extraction Reagent, Thermo Scientific, Waltham, MA, USA) containing protease and phosphatase inhibitor cocktail (100× Halt protease and phosphatase inhibitor cocktail, Thermo Scientific). Subsequently, the brain tissue was homogenized and centrifuged at 10,000 rpm for 10 min to obtain supernatant. The protein concentration levels in each supernatant were measured using a BCA Protein Assay Kit (Thermo Scientific) according to the manufacturer’s instructions. IL-1ß, IL-6, TNF-α, MCP-1, E-selectin, and P-selectin levels in the lysates were examined using high-sensitivity ELISA kits (Quantikine^®^ ELISA, R&D Systems Inc., Minneapolis, MN, USA) according to the manufacturer’s instructions. Briefly, samples were added to the assay plates at 50 μL/well and incubated for 2 h at room temperature. After washing the assay plates with wash buffer, mouse IL-1ß, IL-6, TNF-α, MCP-1, E-selectin, and P-selectin conjugates were added to each well and incubated for 2 h. The reaction was stopped, and the absorbance of each well was read at 450 nm using a microplate reader.

### 2.5. BBB Permeability Assay

BBB permeability was measured using Evans blue [33,34]. Mice were injected with 2% Evans blue (Sigma) solution diluted in normal saline (200 μL) via the jugular vein. After 30 min of Evans blue circulation, the mice were sacrificed and perfused with normal saline, and the brain was harvested. For measurement, Evans blue in the brain tissue was extracted with formamide at 55 °C overnight. On the next day, extracted Evans blue was measured at 620 nm using a microplate reader.

### 2.6. Statistical Analyses

Statistical analyses were performed using Prism v7.0 (GraphPad Software, San Diego, CA, USA). Among-group comparisons were performed using a one-way analysis of variance, followed by Tukey’s post hoc tests for multiple comparisons. Results are expressed as the mean ± standard mean error. Statistical significance was set at *p* < 0.05.

## 3. Results

### 3.1. Noise-Exposure-Induced Neurobehavioral Change under Inflammatory Conditions

**Figure 2:**
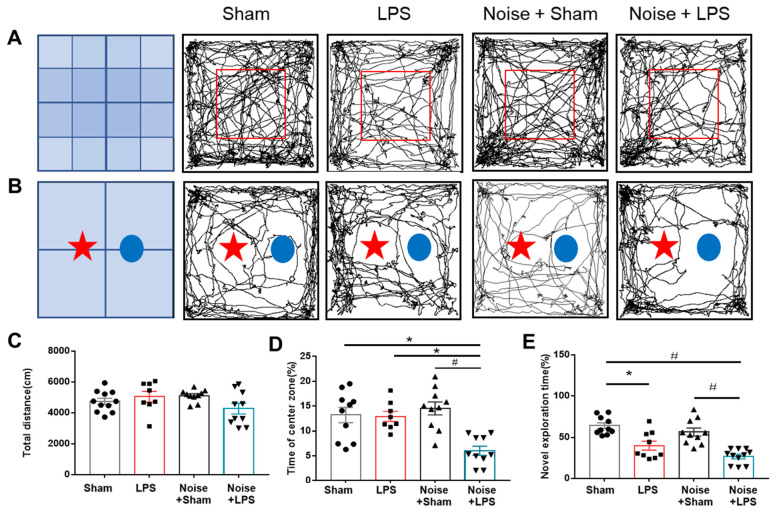
Assessment of mouse neurobehavioral function. (**A**) Representative trajectory movement of mice in (**A**) OFT and (**B**) NORT. (**C**) Total distance and (**D**) percentage of time in the center zone during OFT. The LPS-treated + noise exposure group had a significantly shorter staying time rate compared with the sham control group and noise + sham group (*n* = 8~11 per group) (*p* < 0.05). (**E**) Discrimination time rate for novel objects in the post-trail of NORT. Discrimination time rate was significantly (*p* < 0.05) decreased in the LPS-treated + noise exposure group compared to the sham control group and sham + noise exposure group (*n* = 8~11 per group). * *p* < 0.05 and # *p* < 0.001. LPS, lipopolysaccharide; OFT, open-field test; NORT, novel objective recognition test.

Neurobehavioral tests (OFTs and NORTs) were performed to determine the effect of chronic noise exposure under inflammatory conditions. In the OFT, the total distance did not show significant difference among groups (Figure 2C). However, the percentage of time in the center zone was significantly reduced in the LPS-treated + noise exposure group compared to the sham control group (*p* = 0.001, Figure 2D).

Cognitive function was assessed using the NORT (Figure 2E). The LPS-treated group showed a significantly decreased exploration rate for novel objects compared to the sham control group (*p* = 0.002, Figure 2E). Noise exposure did not alter novel object exploration rates. However, the LPS-treated + noise exposure group showed a significantly decreased exploration rate compared to the sham control and noise exposure groups (*p* < 0.001, Figure 2E). These findings suggest that LPS-induced inflammation could induce cognitive impairment, and noise stress combined with systemic inflammation could induce cognitive impairment and anxiety-like behavioral changes.

### 3.2. Noise Stress Combined with Systemic Inflammation Exacerbates Neuroinflammation

**Figure 3:**
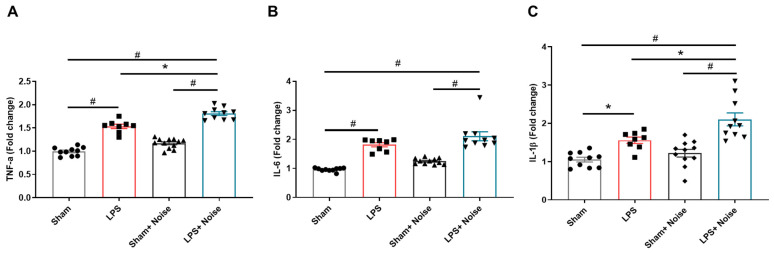
Comparison of pro-inflammatory cytokines in hippocampal tissue. The level of (**A**) TNF-α, (**B**) IL-6, and (**C**) IL-1β was measured in 4 groups (*n* = 8~11 per group). The noise exposure group showed significantly increased levels of TNF-α, IL-6, and IL-1β compared to the sham group (*p* <0.05). The LPS-treated + noise exposure group showed significantly increased TNF-α and IL-6 levels compared to the LPS group. * *p* < 0.05 and # *p* < 0.001. LPS, lipopolysaccharide.

Inflammatory cytokines, including TNF-α, IL-6, and IL-1β, at the hippocampus were assessed using ELISA (Figure 3). The LPS-treated groups showed increased TNF-α, IL-6, and IL-1β levels compared to the sham control group. The LPS-treated groups + noise exposure group also showed increased TNF-α, IL-6, and IL-1β levels compared to the sham control group. Interestingly, the LPS-treated + noise exposure group showed significantly increased TNF-α (*p* = 0.001, Figure 3A) and IL-1β (*p* = 0.014, Figure 3B) levels compared to the LPS-treated group. Also, the LPS-treated + noise exposure group showed significantly increased TNF-α, IL-6, and IL-1β expression levels compared to the noise exposure group (*p* < 0.001, Figure 3). These results suggest that noise exposure combined with LPS treatment aggravated neuroinflammation compared to noise exposure or LPS treatment alone.

### 3.3. BBB Integrity Is Disrupted under Noise Stress Conditions

**Figure 4:**
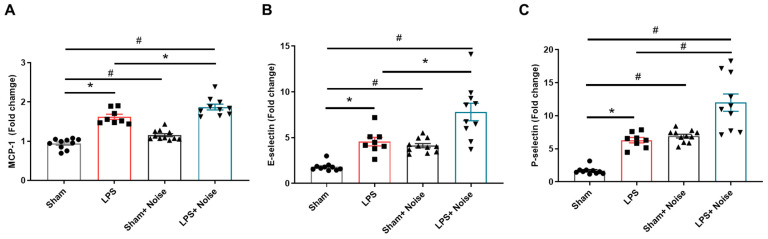
Comparison of proteins associated with BBB disruption in hippocampal tissue. Levels of (**A**) MCP-1, (**B**) E-selectin, and (**C**) P-selectin were measured in 4 groups (*n* = 8~11 per group). The noise group and LPS-treated group showed significantly increased MCP-1, E-selectin, and *p*-selectin levels compared to the sham control group, (*p* < 0.05). The LPS-treated + noise exposure group showed significantly increased MCP-1, E-selectin, and P-selectin levels compared to the LPS-treated group (*p* < 0.05). *p < 0.05 and #*p* < 0.001.

**Figure 5:**
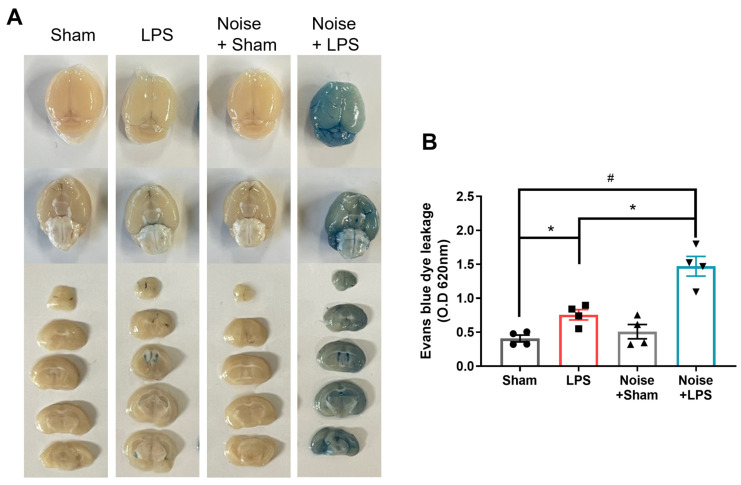
Evaluation of BBB permeability. (**A**) Representative whole brain and coronal sectioned brain images of Evans blue extravasation in the various groups and (**B**) quantitative analysis of Evans blue leakage (*n* = 4 per group). The remaining groups showed significantly increased Evans blue extravasation (*p* < 00.5) compared to the sham control group. The LPS-treated + noise exposure group showed significantly increased Evans blue extravasation compared to the LPS-treated group (*p* < 0.05) (*n* = 3). *p < 0.05 and #*p* < 0.001.

We measured proteins related to BBB disruption, including MCP-1, E-selectin, and *p*-selectin levels, using ELISA to assess BBB integrity disruption (Figure 4). Additionally, BBB disruption was directly evaluated using Evans blue injection (Figure 5). All three groups showed increased MCP-1, E-selectin, and P-selectin levels compared to the sham control. Interestingly, the LPS-treated + noise exposure group showed significantly increased MCP-1 (*p* = 0.021, Figure 4A), E-selectin (*p* = 0.002, Figure 4B), and P-selectin (*p* < 0.001, Figure 4C) levels compared to the LPS-treated group. Evans blue analysis of BBB disruption revealed the LPS-treated group showed impaired BBB integrity compared to the sham group. Moreover, this impaired BBB integrity was much more severe in the LPS-treated + noise exposure group compared to the LPS-treated group (*p* = 0.022, Figure 5B). Our results show that noise exposure under systemic inflammatory conditions can worsen disrupted BBB integrity.

## 4. Discussion

In this study, we investigated the mechanism underlying the development of cognitive impairment in an animal model of septic encephalopathy and environmental noise exposure mimicking the ICU environment. Our findings demonstrate that 75 dB mechanical noise exposure without LPS injection slightly increased inflammatory responses and proteins related to BBB disruption, which did not lead to cognitive impairment. The group treated with LPS but without noise exposure displayed slightly increased inflammatory responses and BBB disruption and revealed cognitive impairment. Interestingly, the LPS-treated + noise exposure group exhibited behavioral changes, including not only cognitive impairment but also anxiety-like behavior. Moreover, the LPS-treated + noise exposure group exhibited an increased neuroinflammatory response and disrupted BBB integrity compared to the group treated with LPS but without noise exposure. These results suggest that continuous mechanical noise exposure under systemic inflammatory conditions worsens the neuroinflammatory response and disrupts BBB integrity more so than systemic inflammatory conditions.

The impact of constant environmental noise on brain function, especially cognitive and memory function, remains unclear, and its adverse effects on brain health are often overlooked. Critically ill patients are exposed to constant noise because ICU beds lack sufficient sound insulation. Indeed, our previous clinical study confirmed that the median noise exposure level (dBA) of all ICU units was measured at 54.4 dB (51.1–57.5) over a 24 h period. It was also observed that patients’ monitoring devices generated a louder noise at 85 dB. There was no difference between day and night, and consistent levels of mechanical noise ranging from a minimum of 41 dB to a maximum of 91 dB were measured in all ICU units. These findings confirmed that continuous exposure to mechanical white noise series adversely affects patient recovery [5]. This could be the reason for the common occurrence of delirium in the ICU [2]. This is consistent with our results that suggest that noise exposure combined with systemic inflammation induces delirium-like behavior, including increased anxiety and cognitive impairment, in an animal model.

According to previous studies, even lower-level noise exerts a slight stress effect and substantially impairs hippocampus-related learning and memory by altering the plasticity of synaptic transmission [35,36,37]. Additionally, high-level noise exposure (123 dB) impairs spatial learning and memory and is associated with decreased hippocampal neurogenesis in mice [38], and chronic white noise exposure has detrimental cognitive effects, including increased anxiety-like behavior in rats [39]. Another animal study involving noise exposure for 5.5 h a day for 21 consecutive days confirmed that increased plasma corticosterone levels may promote stress-related biological negative effects in a noise exposure group, suggesting that external harmful auditory stimuli could affect immune function [40]. Furthermore, chronic environmental noise exposure produces persistent non-auditory disorders that modify hippocampal cell proliferation [41], and long-term noise exposure can accelerate cognitive dysfunction, amyloid-β deposition, and tau hyperphosphorylation in different brain regions, including the hippocampus and cortex [42]. These previous studies have demonstrated that noise exposure can act as an obvious risk factor for cognitive impairment and brain dysfunction.

Neuroinflammation is a major pathogenetic factor for the development and progression of cognitive dysfunction superimposed on CNS disease [43]. We observed that noise exposure under LPS treatment induced increased levels of pro-inflammatory cytokines in the hippocampus and neurobehavioral changes, including cognitive impairment and anxiety-like behavior. Also, previous studies have shown that neuroinflammation blockade ameliorates cognitive impairment [44]. It has been revealed that proteins associated with systemic inflammation, such as ICAM-1, IL-1ß, TNF- α, IL-8, and MCP-1, can traverse the BBB and induce neuroinflammation, which is related to BBB disruption [45]. Indeed, BBB dysfunction has been reported in many CNS pathological conditions, including Parkinson’s and Alzheimer’s diseases, brain tumors, and epilepsy [46,47,48]. Under normal physiological conditions, the BBB plays a crucial role in the brain’s metabolic activity and neuronal function. Thus, maintaining BBB functional and structural integrity is critical to maintaining brain homeostasis and function [49]. A diverse array of molecules, including essential nutrients, immune cells, and cytokines, are transported across the disrupted BBB in pathologic conditions. Therefore, we hypothesized that exposure to environmental noise under inflammatory conditions would worsen cognitive impairment via exaggerated BBB disruption and neuroinflammation. In this study, there was no significant increase in anxiety-like behavior in the LPS treatment group without noise exposure. However, noise exposure under systemic inflammation conditions led to cognitive dysfunction and anxiety-like behavior in addition to an increased neuroinflammatory response and disrupted BBB integrity compared to LPS treatment without noise exposure. However, we could not compare the severity of memory dysfunction between the LPS group and LPS + noise group with the NORT. 

The current study design has limitations, as LPS dosage in this study induced severe systemic inflammation. It is well known that LPS is commonly used to model systemic inflammation associated with neuroinflammation. When injecting LPS into animals, several factors need to be considered, such as the concentration of LPS, the genetic background of the animal, the diet, and the injection method. [50,51]. In order to establish a sepsis encephalopathy mouse model for the current study, we thoroughly investigated the literature on sepsis encephalopathy mouse models using LPS administration. Many previous studies have administered LPS at dosages ranging from 3 to 70 mg/kg to create a sepsis mouse model [25,26,27,28,29,30]. Additionally, our previous study demonstrated that 20mg/kg of LPS administration (i.p.) induces systemic inflammation and neuroinflammation in mice [52]. Administrating LPS at higher concentrations than the range of dosages used to create sepsis models can lead to septic shock, characterized by hypotension and multiple organ failure, and finally to death [51]. Based on these investigations, we decided to administer 5 mg/kg of LPS to mice. However, as the LPS dosage in the current study induced severe systemic inflammation, we failed to determine the additive effect of noise under LPS-induced inflammatory conditions on memory function via the NORT. Therefore, it is necessary to further investigate this in the future by employing a noise exposure experiment with lower LPS injection doses that induce mild systemic inflammation, but not memory impairment. Alternatively, a more delicate neurobehavior test could be performed to determine the additive effect of noise under LPS-induced inflammatory conditions. However, our results clearly demonstrate that persistent noise exposure in combination with LPS treatment induced more severe neuroinflammation and BBB integrity disruption, which in turn induced behavior changes, including anxiety-like behavior and cognitive impairment. The current study has another limitation, as continuous noise exposure can induce sleep deprivation. Although sleep deprivation is known to be one of the contributing factors to cognitive impairment and delirium, we did not consider or investigate it as a controlled factor. Therefore, we suggest that further studies, taking these factors into considerations, are needed to investigate the mechanisms underlying the development of cognitive dysfunction and delirium in the ICU.

BBB disruption is observed in numerous CNS pathological conditions. MCP-1 expression is closely correlated with monocyte infiltration in multiple sclerosis, stroke, and CNS trauma. Previous studies suggest that MCP-1 mediates BBB permeability. Specifically, a high MCP-1 concentration, present in the perivascular space of the BBB, directly induces monocytes infiltration. This suggests that MCP-1 may regulate BBB permeability to facilitate monocyte transmigration during systemic inflammation [53]. Additionally, pathological conditions increase the production of pro-inflammatory cytokines, including TNF-α, IL-6, and IL-1β, as well as MCP-1 [54]. Moreover, an increased inflammatory response induces the overproduction of cell adhesion molecules, including E-selectin, which promote the adherence and trans-endothelial migration of circulating monocytes and other inflammatory cells through the BBB [55,56]. Indeed, we observed increased MCP-1, E-selectin, and P-selectin expression in the LPS-treated + noise group compared to the LPS-treated group in the hippocampal regions in the present study. However, we did not confirm the levels of pro-inflammatory cytokines and MCP-1 in the amygdala region, although Evans blue penetration was observed in the lateral ventricle and amygdala in the LPS-treated + noise group. Taken together, these findings suggest that future studies should confirm the levels of pro-inflammatory cytokines and MCP-1 in the amygdala region to determine the mechanisms of noise exposure under the inflammatory condition associated with anxiety-like behavior. 

## 5. Conclusions

Based on our previous clinical results, the aim of present study was to determine the effects of continuous and controllable levels of noise exposure on postoperative cognitive dysfunction and behavioral changes, and to identify the mechanisms via the mouse model mimicking the ICU environment. In conclusion, our findings demonstrate that noise exposure combined with systemic inflammation aggravated BBB disruption and neuroinflammation in our mouse model mimicking the ICU environment. These findings suggest that constant noise exposure under systemic inflammatory conditions, such as sepsis, may worsen neuroinflammation via severe BBB impairment, which leads to cognitive impairment and anxiety-like behavior. Our findings provide scientific evidence that the physical environment of the ICU should be improved for the brain health of critically ill patients. We propose that regulating persistent, stressful noise exposure for patients under inflammatory conditions is a clinically valuable strategy.

## Data Availability

The data used during the current study are available from the corresponding author upon reasonable request.
